# Evidence of deviant parasympathetic response to social exclusion in women with borderline personality disorder

**DOI:** 10.1007/s00406-023-01678-8

**Published:** 2023-08-31

**Authors:** Eugenia Kulakova, Livia Graumann, An Bin Cho, Christian Eric Deuter, Oliver T. Wolf, Stefan Roepke, Christian Otte, Katja Wingenfeld

**Affiliations:** 1 Department of Psychiatry and Neurosciences, Charité – Universitätsmedizin Berlin, Campus Benjamin Franklin, Hindenburgdamm 30, 12203 Berlin, Germany; 2 Department of Cognitive Psychology, Institute of Cognitive Neuroscience, Ruhr University, Bochum, Germany

**Keywords:** Borderline personality disorder, Social exclusion, Cyberball, Need threat, Autonomous nervous system, Parasympathetic nervous system, Heart rate variability, Vagal tone

## Abstract

Stressful social situations like social exclusion are particularly challenging for patients with borderline personality disorder (BPD) and often lead to dysfunctional reactive behaviour of aggression and withdrawal. The autonomous signature of these core symptoms of BPD remains poorly understood. The present study investigated the parasympathetic response to social exclusion in women with BPD (*n* = 62) and healthy controls (HC; *n* = 87). In a between-subjects design, participants experienced objective social exclusion or overinclusion in the Cyberball task, a virtual ball-tossing game. Need threat scores served as individual measures of perceived exclusion and the resulting frustration of cognitive–emotional needs. Five-minute measurements of high-frequency heart rate variability (HF-HRV) at three time points (before, during, after Cyberball) indicated parasympathetic tone and regulation. We observed a trend towards lowered baseline HF-HRV in BPD vs. HC in line with previous findings. Interestingly, the parasympathetic response of patients with BPD to objective and perceived social exclusion fundamentally differed from HC: higher exclusion was associated with increased parasympathetic activation in HC, while this autonomic response was reversed and blunted in BPD. Our findings suggest that during social stress, the parasympathetic nervous system fails to display an adaptive regulation in patients with BPD, but not HC. Understanding the autonomous signature of the stress response in BPD allows the formulation of clinically relevant and biologically plausible interventions to counteract parasympathetic dysregulation in this clinical group.

## Introduction

Borderline personality disorder (BPD) is a complex mental illness which affects approximately 1–2% of the adult general population and is associated with a high burden of disease [1, 2]. Fear of abandonment and instable interpersonal relationships constitute its central and most debilitating symptoms [3, 4]. Patients with BPD show heightened sensitivity to cues of potential social rejection and, accordingly, higher expectations of negative evaluations and social exclusion [5, 6]. Perceived exclusion leads to an immediate aversive physiological and emotional distress response [7]. This, in turn, activates dysfunctional compensatory behaviour, further straining interpersonal relationships and, in a vicious circle, often promoting actual rejection [8].

A well-established experimental paradigm to study social exclusion is the Cyberball task [9], a virtual ball game in which co-players direct varying amounts of ball tosses towards the participant, thereby including or excluding them from the game. Research employing the Cyberball task shows that patients with BPD readily feel excluded in situations which are objectively including, and react with stronger cognitive–emotional need threat and negative affect [6, 10–14].

An adaptive reaction to psychosocial stress such as social exclusion is the display of prosocial *tend-and-befriend* behaviour aimed at repairing potential interpersonal damage and re-establishing social cohesion [15, 16]. Healthy control (HC) participants who are excluded during Cyberball demonstrate prosocial behaviour by increasing ball tosses towards the excluding partner, increased cooperativity and empathetic concern [17–19]. In contrast, in patients with BPD psychosocial stress often leads to reactive *fight-of-flight* behaviour, which is characterised by the inhibition of prosocial behaviour, lowered empathy and aggressive action tendencies [12, 20]. Due to the biased perception of social participation in BPD, an *over*inclusion condition has been established as the preferred control condition in the Cyberball task as opposed to equal inclusion to overcome exclusion hypersensitivity [14, 21].

While the (top-down) cognitive mechanisms underlying biased sensitivity to social exclusion in BPD are becoming increasingly understood [12, 22, 23], the (bottom-up) physiological and autonomous processes that accompany social exclusion in BPD only recently started to gain attention [10, 14, 24, 25]. One possible link between social behaviour and autonomous physiological function has been suggested by polyvagal theory [26]. According to polyvagal theory, the myelinated ventral vagus nerve—the main nerve of the parasympathetic nervous system—is centrally involved in regulating a social engagement system in order to suppress phylogenetically older defensive *fight-or-flight* reactions driven by the sympathetic nervous system. This *vagal brake* can be seen as the parasympathetic mechanism enabling emotional self-regulation and prosocial engagement and thus a plausible mechanism underlying *tend-and-befriend* behaviour [27]. In recent years, measures of heart rate (HR) and heart rate variability (HRV), which tap into vagal activity, have moved into the focus of clinical research to bridge cognitive–emotional processes with their underlying autonomous regulatory mechanisms. Respiratory sinus arrhythmia (RSA) and high-frequency power (HF) HRV have been demonstrated as the most reliable HRV measures of vagally mediated regulatory capacities, i.e. parasympathetic function [28].

In line with polyvagal theory, several studies identified positive relationships between resting vagal tone and the capacity for positive affect, self-reported empathy, attachment security, emotion regulation, but also attentional control, executive function and inhibitory capacity [29–32]. Furthermore, investigations of vagal reactivity, that is the momentary changes in vagal tone during a stressful task, show that the ability to self-regulate and engage socially was associated with changes in vagal tone [31, 33]. Unsurprisingly, individuals with BPD exhibit lower vagal tone [34, 35] and aberrant HR and HRV reactivity to psychosocial stress [36].

To our knowledge, until now only one study investigated vagally mediated reactivity to Cyberball-induced social exclusion in patients with BPD [37]. The authors reported decreased RSA values during both inclusion and exclusion phases of the Cyberball task in patients with BPD, while RSA was not affected by Cyberball conditions in HC or depressed patients. However, this study had a relatively small sample size and induced social inclusion and exclusion in quick succession within the same participant, session and order. Such within-subjects design might have blunted the physiological response to the different social situations and underestimated the vagal capacity to differentiate between Cyberball conditions. This possibility is supported by studies which do report increased HR and HRV reactivity during Cyberball-induced social exclusion in HC [38, 39]. Furthermore, as mentioned above, the biased perception of inclusion in patients with BPD makes the overinclusion condition of the Cyberball task a more suited control condition to study ostracism in this clinical group.

### The present study

The present study sets out to study the vagally mediated physiological response to social exclusion in patients with BPD compared to a tightly matched group of healthy controls (HC). In a between-subjects design, participants were randomised to either the exclusion or overinclusion condition of the Cyberball task. The HF-HRV was used as the preferred and established measure of vagal activity. We report both vagal tone and vagal reactivity as measures of baseline regulatory capacity as well as acute (during Cyberball) and delayed (after Cyberball) vagally mediated regulatory effort.

Previous research shows that the way patients with BPD perceive Cyberball-induced need threat does not always correspond with the objective extent of social exclusion or (over)inclusion during the game. We were therefore interested in how the vagally mediated regulation response was influenced by the underlying emotional–cognitive factor of need threat (threat to fundamental social needs), which served as an individual measure of perceived ostracism across Cyberball conditions.

We expected to replicate the findings of decreased vagal tone in patients with BPD compared to HC [35, 37]. Furthermore, we expected to observe a vagal response pattern matching the previously demonstrated *fight-of-flight* reaction of patients with BPD during (perceived) social exclusion, while we expected the opposite pattern for HC as indicative of the more adaptive *tend-and-befriend* strategy.

## Methods

### Participants

The sample consisted of 62 female patients with BPD and 87 female healthy controls (HC). Here, we report the results for those participants described by Graumann et al. [11] for which HRV data were collected. Native German speakers between the ages of 18 and 55 with a BMI between 17.5 and 30 were included and underwent the Structural Clinical Interview for DSM-5 Disorders (SCID) (German versions of SCID-5-CV, SCID-5-PD) [40]. Exclusion criteria were neurodegenerative, metabolic, endocrine, autoimmune and CNS diseases, severe somatic diseases, glucocorticoid intake and pregnancy. Additional exclusion criteria for the BPD group were acute major depressive episode, lifetime schizophrenia and other psychotic disorder, substance use disorder, acute suicidal behaviour and the daily intake of more than three different psychotropic substances. HC needed to be free of lifetime psychiatric diagnoses, treatment and medication. HC and BPD groups were matched for age, education, intake of hormonal contraception and menstrual cycle phase. All participants received verbal and written information and gave written informed consent before participation. Participants were reimbursed with 60–90€, depending on their performance in a computer-based task. The procedures were in line with the Declaration of Helsinki and approved by the local ethics committee.

### Procedure and task

The study involved two testing sessions of 1.5 h each. In the first session, participants received diagnostic interviews by trained clinicians and filled out computer-based self-report diagnostic measures using the RedCap online database. In the second session, participants underwent a Cyberball task, which was used to induce social exclusion [9]. Participants were wearing a heart rate belt (Polar H9 sensor) coupled with a Polar V800 watch recording R–R intervals. HRV measurements of interest were continuous 5-min intervals collected before (−25 min), during, and after (+ 50 min) Cyberball in a seated upright position with eyes open.

In the beginning of the session, participants took a quiet seated position for ten minutes, the last 5 min of which served as the HRV baseline measure. Twenty-five minutes later, participants received written instructions for the Cyberball game, a virtual ball-tossing game with two co-players. They were randomly assigned to either the exclusion or the overinclusion condition of the task. Both Cyberball conditions consisted of 30 ball tosses. In the exclusion condition, participants received the ball twice within the first six tosses, but then never again. In the overinclusion condition, participants received the ball in 45% of all tosses, i.e. 13 times. Before the game, participants were told a cover story of two real co-players connected to the game via internet, while in fact the co-players were computer generated. All participants were debriefed at the end of the second session. After receiving the instructions, participants started the Cyberball game. The task lasted around three minutes, during which HRV was continuously recorded. After the Cyberball task participants remained quietly seated and gave saliva samples, completed computer-based tasks and filled out the Need Threat Questionnaire (NTQ) and other questionnaires (see Graumann et al. [11] for details). Fifty minutes after Cyberball, participants took a resting position for the third HRV measurement.

### Need Threat Questionnaire and estimated ball possession

Cyberball-related need threat was assessed with the German version of the Need Threat Questionnaire (NTQ) [41]. On a five-point Likert scale (1 = not at all, 5 = completely), participants indicated their agreement with 14 statements corresponding to four different scales: belonging (e.g. “I felt rejected”), control (e.g. “I felt powerful”), self-esteem (e.g. “I felt popular”) and meaningful existence (e.g. “I felt non-existent”). Following Gutz et al. [12], the sum score of all four subscales was used as the measure of total need threat (range 4–20), with higher values indicating more need threat, that is stronger perceived violation of social–cognitive–emotional needs. Additionally, participants were asked to estimate the percentage of received ball tosses during the Cyberball game.

### Heart rate variability measure

HRV was collected at three time points: (1) at baseline (t0; 25 min before Cyberball); (2) during Cyberball (t1); and (3) after social exclusion (t2; 50 min after Cyberball). For comparability, 5-min intervals (last 5 min of 10-min resting intervals and full Cyberball duration) were extracted and submitted to further analyses. Data were processed with the Kubios Premium software [42]. Intervals were selected manually from the continuous recording and underwent automatic artefact correction (medium threshold setting). This led to the correction of 0.7% of all heartbeats in t0, 0.6% in t1 and 0.6% in t2. Absolute high-frequency (HF) power (0.15–0.4 Hz) was calculated using fast Fourier transformation (expressed in ms^2^). HF-HRV represents the activation of the parasympathetic system [43], and its preferential use is advocated as based on well-understood neurophysiological mechanisms of vagal activity [28, 33]. Because HF-HRV measures violated normal distribution, log-transformation (ln) was performed. We also calculated the average heart rate (HR; expressed in bpm) of the intervals used for HF-HRV calculation. Outlier correction based on baseline HR values was performed to exclude extreme values (± 3SD, corresponding to an included range of 44–112 bpm). This led to the exclusion of two participants (both from the BPD overinclusion condition). Statistical analyses were performed using SPSS version 26.0 [44]. To compare the two groups (BPD vs. HC) with respect to vagal tone, we report baseline measures (t0) of HF-HRV as well as HR, R–R intervals, systolic and diastolic blood pressure. For the central analysis of vagal reactivity to social exclusion, we calculated HF-HRV difference scores (∆HF-HRV) for the acute (during Cyberball) and delayed (after Cyberball) effects by subtracting baseline values from the respective scores (acute: t1–t0; delayed: t2–t0). For replicability purposes, HRV measures are reported in accordance with the GRAPH recommendations [33, 45].

## Results

### Demographic and clinical data

Groups did not differ in age, years of education, use of hormonal contraception, BMI, phases of menstrual cycle and relationship status. The BDP group included a higher amount of smokers. See Table 1 for details.

**Table 1:** Sample characteristics

Variable	BPD *n* = 60	HC *n* = 87	Statistics	
Age (mean, SD)	27 (7)	28 (7)	*t*(145) = −0.42	*p* = 0.68
Years of school education (mean, SD)	12 (1)	12 (1)	*t*(145) = −0.75	*p* = 0.45
Hormonal contraception (y/n)	9/51	14/73	*χ*^2^(1) = 0.03	*p* = 0.86
Smoker (y/n)	22/38	12/75	*χ*^2^(1) = 10.45	*p* < 0.001***
Body mass index (mean, SD)	22 (3)	22 (2)	*t*(145) = 0.77	*p* = 0.44
Cycle phase (follicular/luteal/ no natural cycle)	16/33/11	28/43/16	*χ*^2^(2) = 0.58	*p* = 0.75
In a relationship (y/n)	11/49	22/65	*χ*^2^(1) = 0.99	*p* = 0.32

*BPD* Borderline personality disorder, *HC* healthy controls, *n* sample size, *SD* standard deviation, *y* yes, *n* no

Asterisks indicate significant effects: *p < 0.05, **p < 0.01, ***p < 0.001

In the BPD group, 26 women were inpatients and 34 were outpatients. The following comorbid diagnoses were determined: PTSD *n* = 16, eating disorder *n* = 10, alcohol abuse *n* = 3, drug abuse *n* = 5, agoraphobia with panic disorder *n* = 3, social phobia *n* = 3, panic disorder *n* = 1, obsessive compulsive disorder *n* = 2 and agoraphobia *n* = 1. Overall, 28 women with BPD reported intake of psychotropic medication, and the remaining 32 patients were free of psychotropic medication. Eighteen patients with BPD took one substance, six took two, and four took three different substances. Patients reported taking the following substances: selective serotonin reuptake inhibitors (SSRI) *n* = 15, anti-psychotics *n* = 7, serotonin and noradrenaline reuptake inhibitors (SNRI) *n* = 7, dopamine and noradrenergic reuptake inhibitors (NDRI) *n* = 4, tricyclic antidepressants *n* = 2, noradrenergic and specific serotonergic antidepressants (NaSSa) *n* = 2, anticonvulsants *n* = 1, alpha/beta adrenergic blockers *n* = 1 and methylphenidate *n* = 1.

### Physiological baseline differences between groups

As presented in Table 2, the BPD group showed a higher baseline HR compared to HC. Consistently, R–R intervals were shorter in BPD compared to HC. Neither systolic nor diastolic blood pressure differed between groups. There was a trend towards a group difference in vagal tone, with marginally lower baseline HF-HRV values in BPD compared to HC.

**Table 2:** Physiological baseline measures by group

Variable	BPD	HC	Statistics	
Resting heart rate (bpm)	81 (10)	76 (10)	*t*(145) = 2.83	*p* < 0.01**
R–R interval (ms)	756 (101)	808 (117)	*t*(145) = −2.77	*p* < 0.01**
Systolic blood pressure (mmHg)	112 (10)	112 (11)	*t*(145) = 0.37	*p* = 0.74
Diastolic blood pressure (mmHg)	72 (9)	71 (9)	*t*(145) = 0.30	*p* = 0.73
HF-HRV (ms^2^)	6.15 (1.07)	6.47 (1.11)	*t*(145) = −1.71	*p* = 0.09

*BPD* Borderline personality disorder, *HC* healthy controls

Asterisks indicate significant effects: *p < 0.05, **p < 0.01, ***p < 0.001

### Effect of Cyberball on estimated ball possession and need threat

**Fig. 1:**
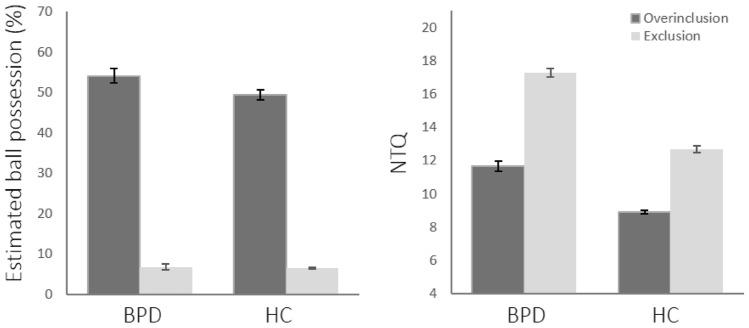
Estimated percentage of ball possession and need threat scores across groups and Cyberball conditions. Estimated ball possession was higher for overinclusion vs. exclusion, with no group differences. NTQ values were higher in exclusion vs. overinclusion, and overall higher in BPD vs. HC. A significant interaction effect indicated higher group differences in exclusion vs. overinclusion. BPD = Borderline personality disorder, HC = healthy controls, NTQ = need threat questionnaire. Error bars indicate standard error of mean

The estimated percentages of received ball tosses were subjected to a 2 (group: BPD vs. HC) × 2 (Cyberball condition: overinclusion vs. exclusion) ANOVA. The analysis revealed no effect of group (*F*(1,143) = 1.31, *p* = 0.25), but a strong effect of Cyberball condition (*F*(1,143) = 444.44, *p* < 0.001) with higher values for overinclusion vs. exclusion. The interaction was not significant (*F* < 1). Both groups were able to accurately estimate the amount of ball possession in both Cyberball conditions (see Fig. 1).

Total NTQ values were submitted to a 2 (group) × 2 (Cyberball condition) ANOVA which revealed a main effect of group (*F*(1,143) = 70.93, *p* < 0.001) and Cyberball condition (*F*(1,143) = 114.81, *p* < 0.001). Need threat was overall higher in BPD vs. HC, and higher after exclusion vs. overinclusion. A significant interaction effect (*F*(1,143) = 4.36, *p* < 0.05) suggested that the NTQ score difference between BPD and HC was less pronounced after overinclusion (*t*(74) = 4.88, *p* < 0.001) vs. exclusion (*t*(85) = 7.00, *p* < 0.001), indicating increased exclusion sensitivity in the BPD group.

### Effect of Cyberball on HF-HRV

To investigate the vagal reactivity to social exclusion we calculated a 2 (group: BPD vs. HC) × 2 (Cyberball condition: overinclusion vs. exclusion) × 2 (time: during vs. after Cyberball) repeated measures ANOVA with the HF-HRV difference scores (∆HF-HRV). The rmANOVA revealed a significant main effect of time (*F*(1,143) = 7.72, *p* < 0.01), suggesting that ∆HF-HRV was higher during Cyberball and decreased at the later measurement point. No interactions including the factor time were significant (all *F*s < 1). There was no effect of group (*F*(1,143) = 1.74, *p* = 0.23) nor of Cyberball condition (*F*(1,143) = 1.88, *p* = 0.17). However, a significant group × Cyberball condition interaction emerged (*F*(1,143) = 4.53, *p* < 0.05). To follow up this interaction, we calculated two 2 × 2 ANOVAs for each time point, respectively.^1^

#### During Cyberball

**Fig. 2:**
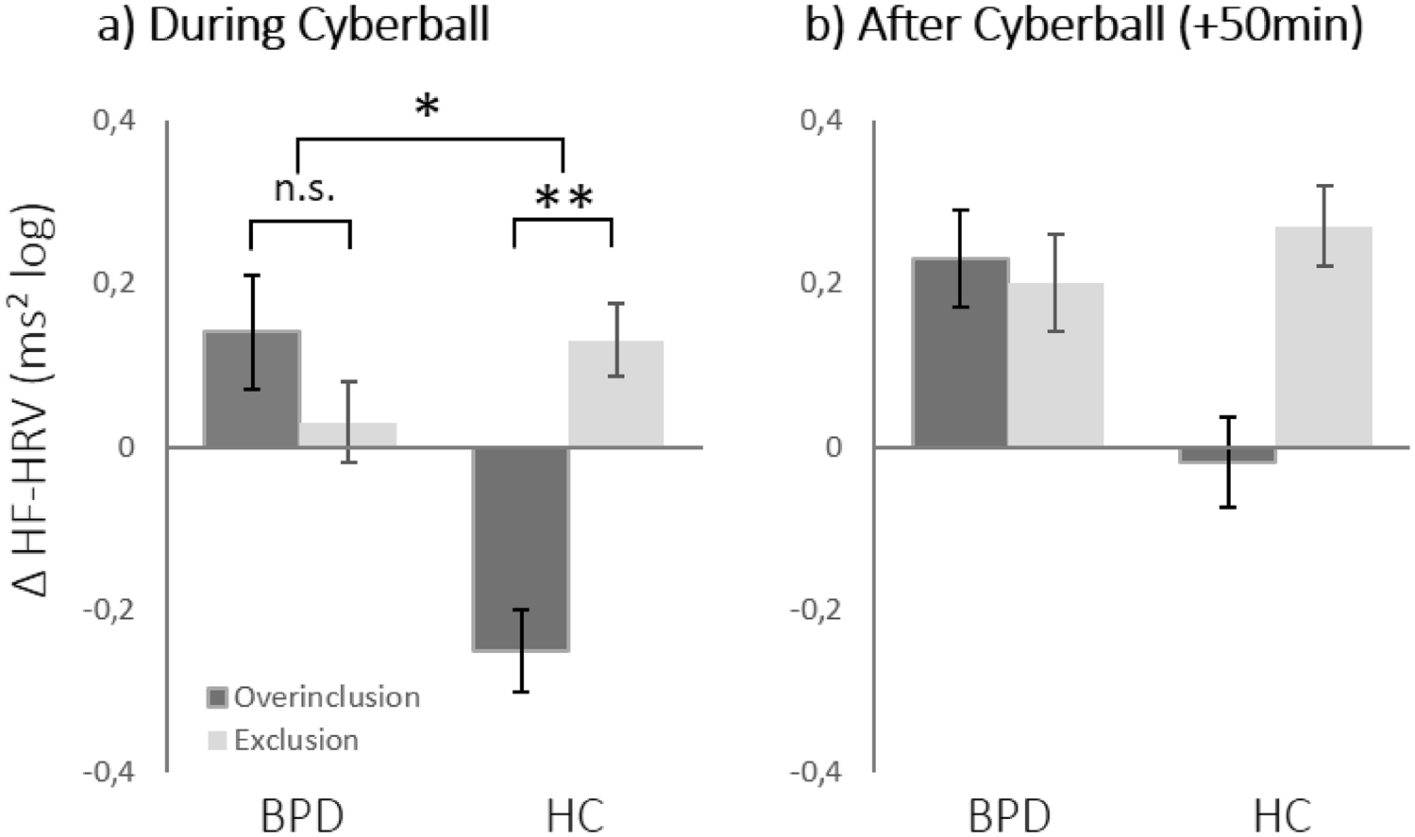
Vagal reactivity (∆HF-HRV) across groups, Cyberball conditions and time points. **a** During Cyberball, a significant interaction effect indicated significant ∆HF-HRV differences between Cyberball conditions in HC, with higher reactivity during exclusion vs. overinclusion. In BPD, this effect was reversed and non-significant. **b** After Cyberball, no significant effects were observed. BPD = Borderline personality disorder, HC = healthy controls. Error bars depict standard error of mean

The ANOVA revealed a significant group × Cyberball interaction (*F*(1,143) = 4.97, *p* < 0.05). Neither the effect of group (*F*(1,143) = 1.67, *p* = 0.20) nor of Cyberball condition (*F*(1,143) = 1.47, *p* = 0.23) reached significance. Follow-up t-tests showed that while in HC ∆HF-HRV was significantly increased in the exclusion vs. overinclusion condition (-0.25 vs. 0.13; *t*(85) = −2.71, *p* < 0.01), the BPD group showed the reverse pattern with higher ∆HF-HRV in the overinclusion vs. exclusion condition. However, this difference did not reach significance (0.14 vs. 0.30, *t*(58) = 0.66, *p* = 0.51). This suggests that patients with BPD did not show a discriminatory ∆HF-HRV response to the Cyberball conditions as did HC, while the slopes of the discriminatory functions between groups were reversed (see Fig. 2a).

#### After Cyberball

Fifty minutes after Cyberball, ∆HF-HRV did not show any significant effects anymore. Both factors of group (*F*(1,143) = 0.64, *p* = 0.42) and Cyberball condition (*F*(1,143) = 1.32, *p* = 0.25) as well as their interaction (*F*(1,143) = 2.07, *p* = 0.15) remained non-significant (see Fig. 2b).

### Association between HF-HRV and need threat

**Fig. 3:**
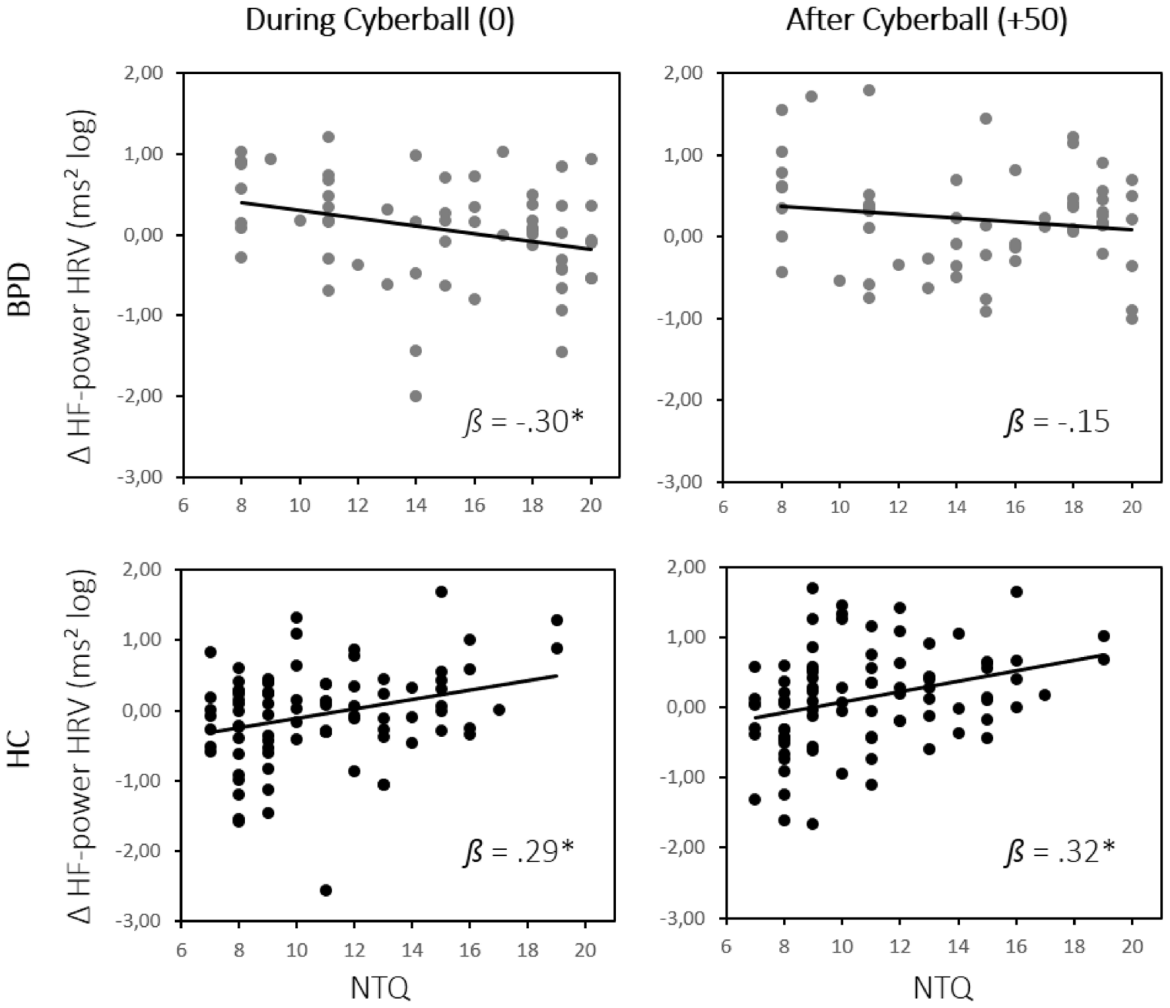
Vagal reactivity (∆HF-HRV) as a function of need threat across groups and time points. While HC showed a positive relationship between NTQ and ∆HF-HRV during and after Cyberball, in patients with BPD increasing NTQ was associated with lower ∆HF-HRV during Cyberball and no significant relationship between the measures after Cyberball. BPD = Borderline personality disorder, HC = healthy controls, NTQ = need threat questionnaire

To specify the vagal reactivity (∆HF-HRV) to social need threat as the cognitive–emotional construct targeted by the Cyberball conditions, we conducted an analysis in which the categorical factor of Cyberball condition was replaced with numerical NTQ scores. A 2 (group: BPD vs. HC) × 2 (time: during vs. after Cyberball) rmANOVA with NTQ scores as a covariate revealed no main effect of time and no interaction including the time factor (all *F*s < 1.02). A main effect of group (*F*(1,143) = 15.08, *p* < 0.001) and a group × NTQ interaction (*F*(1,143) = 15.68, *p* < 0.001) emerged, while the main effect of NTQ was not significant (*F*(1,143) = 1.66, *p* = 0.20). Post hoc linear regression-based parameter estimates were performed for each time point to follow up the significant group × NTQ interaction. The analysis revealed a significant positive association of ∆HF-HRV and NTQ score both during (*ß* = 0.29, *t*(86) = 2.80, *p* < 0.01) and after (*ß* = 0.32, *t*(59) = 3.11, *p* < 0.01) Cyberball in HC. In BPD, the slopes of this relationship were reversed, with a negative association of ∆HF-HRV and NTQ score during Cyberball (*ß* = −0.30, *t*(59) = −2.36, *p* < 0.05), which decreased after Cyberball (*ß* = −0.15, *t*(59) = −1.16, *p* = 0.25). Thus, while increased need threat during Cyberball was associated with an increase of vagal function in HC, potentially suggesting an adaptive and temporally sustained parasympathetic regulatory mechanism, this pattern was reversed for participants with BPD. Here, increasing need threat was associated with decreased ∆HF-HRV during Cyberball, suggesting reduced regulatory vagal activity with increasing perceived exclusion (see Fig. 3).

## Discussion

The present study investigated the parasympathetic response of women with BPD to Cyberball-induced social exclusion. Our findings show a gradually increasing discrepancy between BPD and HC with regard to perceived social exclusion on different functional levels: On a cognitive-perceptual level, patients with BPD accurately discriminated the amount of received ball tosses during both Cyberball conditions, showing no behavioural deviations from HC (in line with [10, 14]). However, on a cognitive-affective level, patients with BPD showed higher need threat in both social situations (overinclusion and exclusion) compared to HC, which is in line with a negativity bias that makes patients with BPD prone to evaluate social situations as generally more threatening and hostile [46]. Despite overall higher need threat, patients with BPD still rated the exclusion condition as more threatening compared to overinclusion, discriminating the Cyberball conditions as accurately as HC did. Differences in need threat between BPD and HC were even higher in the exclusion vs. overinclusion condition, which replicates previous findings of exclusion hypersensitivity [13, 47]. So far, these findings suggest that BPD is characterised not by a mis*perception*, but a cognitive-affective mis*appraisal* of social participation and inclusion.

Adding the autonomous level of vagal reactivity to the picture, the differences between BPD and HC became even more pronounced. While HC showed increased vagal reactivity to exclusion vs. inclusion, the autonomous signature of the Cyberball conditions was reversed and blunted in patients with BPD. This suggests that the vagal response of BPD patients to stressful social exclusion fundamentally differs from the way HC react to acute social stress. The fact that the implicit physiological response showed a smaller difference between Cyberball conditions in patients with BPD vs. HC contrasts the pattern of both groups’ explicit need threat ratings. As such, in patients with BPD the conscious evaluation of the social situation does not closely correspond with the ongoing parasympathetic response. This is in line with findings of deficient top-down regulation of affective processes in BPD, potentially related to frontal dysfunction [48] and deficient fronto-limbic connectivity [1, 49].

Taking into account the need threat ratings, we observed the same pattern of reversed parasympathetic stress-responses between groups. During Cyberball, increasing need threat was associated with equally increasing vagal response in HC, while patients with BPD showed the opposite relationship: increasing need threat was associated with reduced vagal response. The positive association in HC was still present 50 min after Cyberball, while the BPD group showed no association after the game. This could indicate a slow return to adaptive function, potentially corresponding to the sustained state of alarm after perceived social exclusion in BPD [8].

Regarding baseline vagal tone, our findings of higher heart rate and, accordingly, shorter R–R intervals in patients with BPD vs. HC replicate previous results. Similarly, we observe a trend towards lowered HF-HRV in patients with BPD, which also matches previous findings of lowered parasympathetic tone in this clinical group [35].

Overall, our findings match previously reported differential reactions to objective and perceived social exclusion and extend them to the level of autonomous functioning. We can interpret them according to the *tend-and-befriend* vs. *fight-and-flight* discrepancy [11, 50]. The heightened vagal response of HC seems to adaptively compensate (perceived) social exclusion and activate the vagally mediated social engagement system, allowing a *tend-and-befriend* approach. In contrast, in patients with BPD increased perceived exclusion was associated with a reduction of adaptive vagal function, which might be the autonomic precursor of dysregulated social functioning and the dominance of the phylogenetically older *fight-or-flight* response. This physiological response promotes dysfunctional interpersonal behaviour tendencies that are core symptoms of BPD: aggression or withdrawal. Increasing our understanding of the role of the parasympathetic nervous system offers a promising new level of intervention and potential modification of the resulting physiological and behavioural responses.

Based on our findings, certain clinical implications can be discussed. First, they suggest that patients with BPD can have access to an accurate cognitive representation of a social situation. It is rather the affective evaluation and autonomous regulation during the situation that is deviant. As such, strengthening the internally available trace of cognitive information while reducing reliance on the (biased) affective interpretation can offer biologically plausible coping strategies for patients with BPD during (social) stress. Similar approaches like fact-checking or acting opposite to the emotional urge are successfully implemented regulatory skills promoted in dialectic-behavioural therapy (DBT) for BPD [51]. Based on our findings, adding an external measure of HF-HRV can provide an objective autonomous marker of the success of these skills and measure therapeutic progress, at the same time increasing interoceptive awareness of the ongoing autonomous processes as implemented in biofeedback therapy [52]. Above that, patients with BPD might profit from a general up-regulation of their vagal tone to increase regulatory capacity. Certain mindfulness-based and body-centred techniques have already been established in well-validated therapeutic treatments for BPD such as DBT or mentalisation-based therapy (MBT) [53]. In particular, breathing techniques such as diaphragmatic breathing [54, 55], coherence breathing [56], or increasing the exhalation-to-inhalation ratio [57] have been shown to affect parasympathetic tone. Such techniques might be easily and inconspicuously applied before or during social situations to increase the ability of emotional and interpersonal regulation [58, 59].

Importantly, more research is needed to understand the unique signature of the parasympathetic response in BPD, as our results indicate that it can show the opposite pattern to the one observed in HC. Similarly, self-harm like cutting behaviour—a common but highly maladaptive emotion regulation strategy of patients with BPD—has been shown to *increase* HRV in BPD, in turn *increasing* adaptive fronto-limbic coupling [60, 61]. Clinicians have to be cautious about potentially counterintuitive reactions of patients with BPD and resulting paradoxical effects of interventions that have proven useful in non-clinical samples. More research is also needed to better understand the mechanism of such reversed autonomic patterns in BPD. Interactions with early deviations of physio-endocrinal responses, including the HPA axis and the endocannabinoid system, have been recently discussed [62–65].

### Strengths and limitations

While the large sample size and tight matching between BPD and HC groups is a strength of the present study, the sample only included women with BPD. Future research is needed to extend the findings to all genders, since sex differences in the BPD stress response [66] and autonomous nervous function have been reported [49]. Furthermore, half of the BPD sample were taking psychotropic medication, which might affect HRV. However, most common antidepressants such as SSRIs and SNRIs have been shown to lack any noticeable effect on HRV, while the strongest HRV modulations have been reported for TCA, which were prescribed to only two participants of the previous sample [67, 68].

## Conclusion

Our results suggest that patients with BPD can cognitively appreciate the level to which they are socially included in a situation, and actual exclusion leads to increasing need threat. However, the autonomous reaction to need threat is reversed in patients with BPD compared to HC, leading to the failure to activate an adaptive parasympathetic response that would allow emotional regulation and social engagement. It appears plausible that the fact that this dysregulated response to need threat is realised on the level of the autonomous nervous system contributes to its immediate visceral phenomenology and difficulty to regulate, as often reported by patients [7].

## Footnotes

1 A pooled value of both time points would have lacked a clear interpretation regarding the influence of acute social exclusion.
